# Exploring the process of restoring psychological needs after incidences of frustration and need unfulfillment

**DOI:** 10.3389/fpsyg.2024.1413963

**Published:** 2024-06-04

**Authors:** Birgitte Westerskov Dalgas, Nikos Ntoumanis, Karsten Elmose-Østerlund, Thomas Viskum Gjelstrup Bredahl

**Affiliations:** ^1^Department of Sports Science and Clinical Biomechanics, University of Southern Denmark, Odense, Denmark; ^2^Norwegian School of Sport Sciences, Oslo, Norway

**Keywords:** basic psychological needs, need frustration, need unfulfillment, need restoration, psychological recovery

## Abstract

**Background:**

Research on psychological need restoration after incidences of need frustration holds promise for deepening our understanding of the dynamic nature of psychological needs proposed by self-determination theory. We aimed to extend this work by exploring differences in the process of restoring psychological needs after indences of frustration versus need unfulfillment.

**Methods:**

In-depth semi-structured interviews were conducted with 42 Danish adults varying in age, gender, and physical activity levels. Data were analyzed using the Framework Method.

**Results:**

We identified four distinct yet interconnected phases in the need restoration process: Discrepancies between Actual and Desired Need States, Experiencing Negative Emotions, Initiating Plans for Action, and Action Stage. These stages offer a comprehensive framework for understanding how individuals restore their needs.

**Discussion:**

We discerned contrasting approaches to need restoration depending on prior experiences of need frustration due to external contingencies versus need frustration due to internal factors and need unfulfillment. Need frustration due to external contingencies prompts withdrawal, aligning with the avoidance strategies identified in the literature. Conversely, unfulfilled needs and need frustration due to internal factors lead to proactive engagement, highlighting a distinct ‘fight’ response. These insights extend existing research, providing a nuanced understanding of the dynamic processes of need restoration.

## 1 Introduction

Self-Determination Theory (SDT) is a psychological framework used to understand human motivation across various life domains, including education, work, health, and physical activity (Ryan and Deci, 2017). SDT posits that humans have three basic psychological needs (autonomy, competence, and relatedness). The need for autonomy refers to the need to feel volitional and responsible for one’s own actions. The need for competence is the need to feel effective in interactions within one’s social context and have opportunities to develop one’s abilities. The need for relatedness is the need to feel connected to and accepted by others (Ryan and Deci, 2017). The social environment supports or undermines these needs. Need supportive settings encourage need satisfaction, while need thwarting social environments lead to need frustration (Ryan and Deci, 2017; Bartholomew et al., 2018). Recent research has advocated for a third psychological need state that exists in-between need frustration and need satisfaction, namely need unfulfillment (or dormant need; Reeve et al., 2023). Need unfulfillment describes a state where one feels that their psychological needs are set aside or neglected (Bhavsar et al., 2020; Huyghebaert-Zouaghi et al., 2021; Ntoumanis, 2023). Environments that are indifferent to others’ psychological needs are likely to foster perceptions of need unfulfillment (Huyghebaert-Zouaghi et al., 2023; Ntoumanis, 2023; Reeve et al., 2023).

Autonomy frustration refers to experiences reflecting a sense of being controlled by contingencies outside of the self (e.g., through rewards or perceived obligation). Competence frustration refers to experiences reflecting a sense of non-effectance or lack of mastery, incompetence and lack of skills. Relatedness frustration refers to experiences reflecting a sense of social alienation, exclusion, loneliness and lack of support for personal choice and volition (Chen et al., 2015; Ryan and Deci, 2017; Quested et al., 2021). Autonomy satisfaction refers to experiences reflecting a sense of volition, choice, personal agency, optimal challenge and sense of behavior emanating from the self. Competence satisfaction refers to experiences reflecting effectance, mastery, and opportunities for using and extending skills and expertise. Relatedness satisfaction refers to experiences reflecting connectedness and a feeling of being accepted by others (Ryan and Deci, 2017; Quested et al., 2021). Autonomy unfulfillment refers to experiences reflecting uncertainty, ambiguity, and a lack of purpose or meaning. Competence unfulfillment refers to experiences reflecting a sense of not performing or improving as well as one could. Relatedness unfulfillment refers to experiences reflecting a sense of not fitting in or not having much in common with one’s peers (Huyghebaert-Zouaghi et al., 2021).

The satisfaction and frustration of three basic psychological needs (autonomy, competence, and relatedness) influences the degree to which motivation is autonomous or controlled (Ryan and Deci, 2017; Quested et al., 2021). When need frustration is experienced, the motivation that underpins the behavior is controlled, subsequently leading to negative outcomes, as previously mentioned. Within SDT, motivation is categorized as either autonomous or controlled. Autonomous motivation, considered as high-quality motivation, emanates from an individual’s authentic self and aligns with their values and interests or results from feelings of enjoyment. On the contrary, controlled motivation, deemed as low-quality, is driven by internal or external contingency factors such as feelings of guilt, rewards, or punishments (Ryan and Deci, 2017). When need satisfaction is experienced, the motivation that underpins the behavior is autonomous, subsequently leading to positive outcomes (Ryan and Deci, 2017; Quested et al., 2021). Research has suggested that need unfulfillment is associated with maladaptive functioning, but, it is not as psychologically harmful as experiences of need frustration (e.g., classroom disengagement vs. classroom defiance; Reeve et al., 2023).

However, assuming that frustration of psychological needs uniformly promotes controlled motivation or maladaptive outcomes may be an oversimplification. Individuals often seek ways to positively adapt to their context, employing coping strategies that can mitigate the negative effects of need frustration. Research on need crafting demonstrates that individuals actively seek to restore their psychological needs through various means, which can result in adaptive outcomes even in the face of need frustration (Laporte et al., 2021, 2022).

### 1.1 The need restoration process

In recent years, there has been a growing interest in the need restoration process in the SDT literature. Within the need restoration process literature, it is argued that experiences of need frustration do not lead to passive suffering but activate a restorative process aiming at restoring needs and, subsequently, autonomous motivation (Sheldon and Gunz, 2009; Van Prooijen, 2009; Radel et al., 2011, 2013; Waterschoot et al., 2020). Previous studies focusing on the restoration of the need for autonomy have found that when individuals perceive autonomy frustration, they undergo unconscious cognitive adaptations, such as attentional biases toward autonomy-supporting situations, that predispose them to seek autonomy satisfaction once again (Sheldon and Gunz, 2009; Van Prooijen, 2009; Radel et al., 2011, 2013). A study by Anicich et al. (2020) explored the restoration of autonomy under unique circumstances, examining how full-time employees navigated this process during the global pandemic. This study provided evidence that individuals begin the restoration process immediately following the onset of a stressor and continue to do so as the stressor persists. Furthermore, other studies have found that individuals whose autonomy is frustrated are likely to feel a heightened sense of intrinsic motivation when they subsequently engage in activities that support their autonomy (Radel et al., 2014). This heightened sense of intrinsic motivation is particularly noticeable in individuals who have a high autonomy orientation (Fang et al., 2022). In a similar vein, the restoration of the need for competence has also received scholarly attention. Studies have found that students with high levels of competence frustration were more intrinsically motivated in subsequent courses that could restore their competence (Fang et al., 2017) or sought to restore their competence by engaging in a less challenging subsequent task (Fang et al., 2018, 2021). Restoration of the need for relatedness has similarly been addressed in the literature, revealing patterns that parallel those found in restoration studies focusing on the need for autonomy. Research highlights that socially excluded individuals manifest heightened attentiveness to social inclusion cues. For instance, DeWall et al. (2009) demonstrated that these individuals rapidly identify smiling faces, allocating greater attentional resources to such welcoming stimuli. In addition, Maner et al. (2007) elucidated that when the need for relatedness is frustrated, individuals display a predilection for collaborative endeavors, choosing to engage in tasks with others rather than in isolation. These studies collectively suggest that upon experiencing need frustration, individuals engage in adaptive strategies to restore their psychological needs.

The literature on psychological need restoration has not distinguished between need frustration and unfulfillment, partly because this distinction has only recently been incorporated in the SDT literature. For instance, a laboratory study by Maner et al. (2007) examined the effects of social exclusion on the motivation to form new social bonds. In a series of six experiments with undergraduate students, Maner et al. (2007)found that social exclusion generally increases the desire to connect with new individuals and enhances positive behavior toward them, except toward the excluding agents or where no future interaction is expected. This indicates that individuals tend to withdraw from situations where they experience need frustration, but also that they subsequently become autonomously motivated to restore their needs in subsequent situations if the environment is more supportive. In contrast, Sheldon and Gunz (2009) explored the concept of ‘unmet needs’ and how the absence of need satisfaction can motivate individuals to seek experiences that will fulfill these needs. Their study 3 suggests that these needs are fundamental drivers of behavior; when unmet, they create a motivational force that compels individuals to take action to address the deficiency. While both mentioned studies found that need frustration and need unfulfillment can motivate individuals to take actions that restore their psychological needs, they highlight different pathways for the restoration process.

### 1.2 The current study

While the existing literature has contributed to our understanding of the role of the need restoration process, there are notable limitations that call for further exploration. First, although existing studies have done a commendable job exploring the coping strategies employed after incidences of need frustration (Waterschoot et al., 2020) and the restoration of individual psychological needs in isolation (e.g., Maner et al., 2007; Radel et al., 2011; Fang et al., 2017), they have not discussed how these needs might interact with each other. A more holistic view of the need restoration process could be achieved by investigating these interactions. Second, past research has not distinguished between need frustration and unfulfillment. By distinguishing these states in the context of need restoration, the study could contribute to a more nuanced conceptual understanding of dynamic processes of psychological needs change within SDT. Thirdly, there is a paucity of studies that focus on the restoration process within a real-life setting. Psychological need restoration always take place within a particular life domain. The present study specifically focuses on the domain of physical activity, specifically within the contexts of physical education, exercise and sports. By grounding the study in real-life contexts, the insights generated contribute to conceptual development and carry practical significance (Reis and Gosling, 2010). Lastly, there is a prevailing methodological bias toward quantitative approaches in the existing literature. The incorporation of qualitative methodologies, such as in-depth interviews, would likely enrich our understanding, offering nuanced insights that quantitative methods may not be able to fully capture.

Ultimately, our study aims to make a conceptual contribution to the growing interest in the process of restoration of psychological needs that follow incidences of need frustration or need unfulfillment. A more nuanced understanding of the process of need restoration is important for theory and model refinement within the SDT literature because need restoration pathways might differ depending on whether one has experienced need frustration or need unfulfillment. Also, our study aims to provide more evidence regarding the dynamic nature of psychological needs and move beyond their current treatment as static entities.

## 2 Methods

### 2.1 Participants

We conducted semi-structured life story interviews with 42 Danish adults of various physical activity levels. The participants were drawn from a larger nationwide survey (*N* = 163,000) that investigated their physical activity participation and their perceived opportunities and motivation for physical activity. Utilizing maximum variation sampling (Schreier, 2018), we purposefully selected individuals representing diversity according to physical activity frequency levels, gender, age, and geographic locations within Denmark. Given the participants’ diverse backgrounds, we expected to gather in-depth and varied insights into the processes of restoring their psychological needs, enriching our study’s overall understanding.

Initial contact with the participants was made through email by the first author. She invited them to participate in the study and informed them that the study would focus on motivation for physical activity. Out of 436 email invitations sent, we received 58 positive replies. We reached data saturation after conducting 42 interviews; hence, we ended data collection then. This decision balanced robust, comprehensive findings and optimized time and resources for both researchers and participants.

The sample comprised 16 individuals with low, 14 with medium and 12 with high physical activity frequency. These frequency levels were calculated relative to the responses from the whole dataset (*N* = 163,000) across four domains of physical activity: transport, occupation, household, and recreation. At each of the four domains for physical activity, indexes were calculated, where the respondents were grouped into five equally-sized groups and assigned values from 1 to 5, with 1 representing the least active 20% of the respondents and 5 representing the most active 20%. These indexes were then combined into one summative index across the four domains. The 20% least active respondents, as determined by the summative index, were labeled ‘low’ participation, the mid 60% were labeled ‘medium’ participation, and the highest 20% were labeled ‘high’ participation. The participant pool was balanced in terms of gender, with 19 men and 23 women, and spanned three age groups: 15–29 years (15 participants), 30–54 years (13 participants), and 65 years or older (14 participants). Participants resided in 18 different municipalities within Denmark, varying in geographical location, population density, and average socio-economic status. For further information about the participants, refer to the Supplementary material. The sampling method was applied to ensure varied experiences among participants.

### 2.2 Procedures

The semi-structured interviews were conducted by the first author, a female PhD student with formal training and experience in qualitative research, using a pre-tested guide. The introduction included a briefing where the interviewer explained the purpose of the study, the interview process, and the ethical considerations, including the assurance of confidentiality and the participants’ rights to withdraw at any time. The interview guide was based on SDT and was designed to prompt the exploration of individual experiences of need frustration, unfulfillment, and satisfaction in physical activity contexts while maintaining enough flexibility to adapt to participants’ unique responses and narratives. Utilizing probes, we delved into memorable episodes that characterized their experiences, asking participants to discuss specific instances or narratives. A more detailed description of the interview guide can be found in the Supplementary material.

Participants were individually interviewed once, primarily face-to-face (*n* = 38), while a few interviews were conducted via a digital video platform upon the interviewees’ request (*n* = 4). The interviews lasted between 75 and 180 min (average = 130 min). Interviews were conducted in 2021 (March to December) and were held in various locations to accommodate participants’ preferences and ensure their comfort, including their homes, workplaces, a public park, and a library. The interview conditions were chosen to facilitate a relaxed environment conducive to open and candid discussions. In most cases, only the interviewer and the participant were present to maintain confidentiality and minimize distractions. However, in two instances, participants requested public locations, and in two other instances, participants’ spouses remained in the same room, which might have influenced the dynamic of the interview. For more detailed information, see the Supplementary material.

Before recruiting participants, an Institutional Review Board granted approval (protocol code 10.680 on 08.11.2019). Participants received detailed information about the study’s objectives, methodology, and their involvement, provided both in the email invitation and at the start of the interview. They were then asked to provide consent, with the assurance that they could withdraw from the study at any time without consequences.

### 2.3 Data analysis

The interviews were audio recorded and transcribed verbatim. The transcriptions were imported into the NVivo software (version 20.7.1) for data analysis. After the interviews were conducted, field notes were taken, capturing the context, non-verbal cues, and the researcher’s initial interpretations. For example, a field note detailed a participant’s hesitation or enthusiasm when discussing certain topics, the researcher’s own feelings during the interview, and reflections on the interaction dynamics. The field notes were subsequently integrated into the NVivo software, providing an opportunity for researcher reflexivity by allowing systematic comparison of researcher observations with interview data, enabling critical self-examination of potential biases and assumptions throughout the analysis process. We applied the Framework Method (Ritchie and Spencer, 1994), a specialized form of codebook thematic analysis (Braun and Clarke, 2020, 2021). This analytical method was selected due to its rigorous yet adaptable framework for managing qualitative data. It not only accommodates the early establishment of themes but also allows for their iterative refinement and the emergent identification of new themes throughout the analytical process (Ritchie and Spencer, 1994; Braun and Clarke, 2020, 2021). The five phases of the framework analysis (familiarization, development of the framework, indexing, charting, and interpretation) (Ritchie and Spencer, 1994) provided a structured form of data management that helped us capture patterns across the participant a’sccounts despite the high amount of data. The criteria used for sampling were not considered during the analysis process.

The first author conducted this analysis in dialog with co-authors. First, we familiarized ourselves with the data by thoroughly engaging with the interview recordings and transcripts, documenting initial impressions. Then, we created a codebook consisting of four main code categories, each with corresponding sub-themes. The first section encompassed need frustration, the second need unfulfillment, and the third one need satisfaction. The sub-themes for these three categories included each of the three basic psychological needs – autonomy, competence, and relatedness. The fourth category, restoration adaptations, captured the strategies participants employed to restore their needs after incidences of need frustration and need unfulfillment. By systematically assigning portions of the transcripts to relevant codes from the codebook, we deductively analyzed the data. We charted the coded data into a matrix, with rows representing each participant and columns representing each of the code constructs (refer to the Supplementary material for the code matrix). As we found patterns in the participants’ experiences, the analysis became more inductive. We engaged in iterative refinement of these to accurately capture the nuanced complexities inherent in the restoration process. The scope of these themes was adjusted, either narrowed or broadened, and some were merged or split to offer more precise conceptual clarity. Themes and their names were iteratively reviewed, refined, and revised throughout the writing of this paper.

### 2.4 Ethical considerations

Ethical approval for conducting this study was granted by an Institutional Review Board prior to participant recruitment. Before participating in the study, participants received study details and gave verbal consent, knowing they could withdraw at any time without any negative consequences. Anonymity was ensured through pseudonyms and confidential data storage on a password-protected computer accessible only to the research team.

## 3 Results

We identified four distinct yet interconnected phases in the need restoration process: Discrepancies between Actual and Desired Need States, Experiencing Negative Emotions, Initiating Plans for Action, and Action. The following section presents a description of each of the phases, grounded in SDT and supported by participants’ quotes.

### 3.1 Discrepancies between actual and desired need states

Discrepancies between actual and desired need states served as the starting point for the restoration process. Participants experienced need frustration and unfulfillment by identifying discrepancies between desired and current levels of their needs for autonomy, competence, or relatedness. For example, Laura (aged 17) experienced relatedness frustration when she was demoted to a reserve position in her Team Gym practice. She described her experience as follows:

If you were not good enough, you would be taken off the team and become a reserve […] You are jumping for yourself, and the whole team is jumping together and doing rhythm together. The focus was on the ten girls on the team while I stood on the sideline. (Laura)

Laura viewed her membership in the team as dependent on her performance levels, which put her in a vulnerable position of being sidelined as she did not meet the team’s expectations. During practices, Laura found herself physically separated from the team, literally standing on the sidelines. This spatial isolation underlined her perception of being disengaged from the team’s core activities, including their collective jumping and rhythm exercises. Moreover, Laura perceived that the team’s attention was disproportionately allocated to the 10 active members, reinforcing her sense that she was not a valued part of the group. These various perceptions contributed to her overall feeling of disconnectedness from the team.

This pattern of perceiving discrepancies between actual and desired need states was also evident among participants who experienced other types of need frustration. For instance, Lisbeth’s (aged 69) experience below illustrates how she experienced competence frustration. Lisbeth found school gymnastics challenging, becoming aware of her physical limitations. She described her situation as follows:

We had gymnastics at school [...]. I weighed 5-7 kilos too much and a little more than the others […] Sometimes, we had to lay under the bar, swing the leg, and get ourselves over, and I could hardly do it. It was really difficult. (Lisbeth)

In Lisbeth’s account of her experiences of school gymnastics, three considerations stood out: physical inadequacy, comparative disadvantage, and task difficulty. Lisbeth perceived herself as physically inadequate, underscored by her belief that she weighs 5–7 kilos too much and, therefore, she is not suitable or capable of performing gymnastic activities in physical education. When Lisbeth notes that she weighs “a little more than the others,” she makes a social comparison, where she perceives herself as falling short relative to her peers. The task difficulty comes to the forefront in Lisbeth’s description of the physical struggle in the specific gymnastic exercise of laying under the bar and swinging her leg to get over it. She perceived the activity as highly challenging, almost impossible for her, further reinforcing her opinion of physical inadequacy. This perception of task difficulty interacts with her earlier perceptions of physical inadequacy and comparative disadvantage, compounding her overall sense of not measuring up either to the task at hand or to her peers.

Lene (aged 66) was also experiencing competence frustration stemming from discrepancies between actual and desired need states. However, while Laura and Lisbeth experienced need frustration due to the action of others, that is, external contingencies, Lene experienced need frustration due to internal factors. She noted,

“I have gained a lot of weight. I have gotten a big belly, which I usually do not have. [...] I think it is ugly. I actually think a lot about it […] I have a rowing machine upstairs that I forget to use... I struggle to maintain good intentions and find it hard to stick to a routine.”

Lene’s situation is characterized by a notable disconnect between her desired physical appearance and her actual condition. She recognizes a problem (weight gain, altered physical appearance) and possesses the means to address it (a rowing machine), yet struggles to take consistent action. This discrepancy between her ability to identify what needs to be done and her actual behavior indicates an experience of competence frustration. She feels ineffective and incapable of managing her physical appearance.

The same pattern of experiencing discrepancies between actual and desired need states was evident among those who experienced need unfulfillment. Andreas (aged 16), for example, found himself in a football club environment that was acting indifferent to his psychological needs. He said:

Things were beginning to unravel at my previous club. There were frequent stops for various reasons and a change in the coaching staff […] The new coach was just an older brother to one of the others. The training sessions were limited to technical skills. He just stood there and acted as a coach, pretending to be wise in giving advice. His approach seemed ineffective to me. (Andreas)

Andreas’ account highlights a discrepancy between the actual coaching he received and the comprehensive empowering approach he desired. His experience of the training being limited to technical skills points to an unmet need for competence, indicating that the sessions did not fulfill his aspirations for broader skill development. Furthermore, his perception of the coach’s ineffectiveness suggests a lack of meaningful guidance, as he found the advice to be more of a pretense than genuinely useful. These factors collectively reveal a gap between Andreas’ actual state of need unfulfillment and his desired state of need satisfaction.

### 3.2 Experiencing negative emotions

We frequently observed that participants discussed their emotions alongside their accounts of discrepancies between their actual and desired need states. Specifically, negative emotions served to reinforce their cognitive interpretations of need frustration or unfulfillment. In this way, their negative emotions initialized the effort toward restoring psychological needs by avoiding or withdrawing from the need undermining context. For example, Laura, who experienced relatedness frustration as she was demoted to a reserve position in her Team Gym practice, further noted, “You feel excluded [...] You get really irritated or sad. Like, I want to go home now.” Laura articulated emotions of irritation and sadness. These states directly resonate with her experience of being sidelined in her team activities. Her experience of negative emotions is an indicator of her frustrated need for social inclusion and acceptance by the team. Driven by these emotions, she develops a desire to leave the situation and go home as a way to escape further experiences that could reinforce her sense of not belonging.

This pattern was also evident among participants who experienced other types of need frustration. Lene, who had gained weight and struggled to do something about it, said, “When I see myself in profile in the mirror, I feel so bad.” Lene’s negative emotions, in this context, serves as an indicator of her psychological distress about her appearance. Her emotional response to her physical appearance underscores and amplifies her cognitive recognition of the gap between her desired and actual physical states.

Andreas expressed his negative feelings subtly, stating, “It did not feel right for me.” Another common way of articulating negative emotions in relation to need unfulfillment among the participants was “It was extremely boring,” as expressed by Niels (aged 69), who purchased an exercise bike to enhance his health. These emotions responses, less intense and more reflective, contrast with the intense emotions of Laura (irritation and sadness) and Lene (distress about her physical appearance). Andreas’ and Niels’ diffuse and subdued emotional reaction was characteristic of the state of need unfulfillment, compared to the more intense emotion responses seen in the aforementioned need frustration experiences.

### 3.3 Initiating plans for action

In the Initiating Plans for Action phase, participants developed a plan for action to change their psychological need state. An internal drive to address discrepancies in actual and desired psychological need states was evident in all participants’ narratives. For example, Lisbeth said,” If one could, then one should participate […] However, I certainly did not like it, so if I could avoid it, I would gladly do so.” Her approach, marked by avoidance, reflects a deliberate choice to withdraw from activities that contribute to her experiences of need frustration as a means to restore her psychological needs.

However, the participants’ initiation of plans for action to restore their needs varied. We found that some participants’ development of plans to restore their needs was heavily influenced by social dynamics or their available resources. Laura’s telling illustrates this influence of social connections on decision-making. She said: “It was very demotivating, and then I just lost the desire in the end. Some of the people I talked to also stopped, and then I was like, I do not want to do it anymore.” Her decision to disengage was not made in isolation but was deeply influenced by the actions and decisions of those around her, highlighting the interplay between personal feelings and the social environment. In a parallel manner, Andreas’ decision-making was also significantly influenced by social dynamics, albeit in a different context. An opportunity arose through a personal connection, as he explained: “An old friend of my father’s, who was coaching another club, invited me over. I decided to try to train with them.” This decision was catalyzed by the prospect of a new environment, indicating the role of external opportunities and social connections in his initiative to change clubs. Both Laura and Andreas demonstrate how social dynamics and connections can significantly influence individual decisions during the Initiating Plans for Action phase. While Laura’s decision appears more reactive, influenced by a loss of belonging within her team, Andreas’ decision was proactive, driven by the opportunity to join a new team through a personal connection. This difference might be due to their different need states, with Laura experiencing need frustration and Andreas experiencing need unfulfillment, both due to external contingencies. The influence of social dynamics parallels the tendency to use available resources for the initiation of plans for action observed in other participants. Hanne’s case exemplifies this. Confronted with her own perceived limitations in tennis, she leveraged the skills of the ball boys, who also offered tennis coaching. Hanne reflected, “Well, I never became really good at it [...] But there were those ball boys running around collecting the balls [...] They taught tennis if you paid them a little for it, and they got me to a certain level where I could serve and return a ball and learn a bit. So I could keep up when people played.” This approach of seeking instruction enabled her to enhance her tennis abilities and, consequently, her perceived competence. Her strategy of making incremental adjustments to her environment through utilizing available coaching resources underlines how participants tapped into accessible support systems to develop action plans.

For other participants, the initiation of plans for action was a reflective process marked by internal deliberation. Take, for example, the case of Lene, who shared:

I think a lot about doing something to take care of myself and get started. I want to focus on exercise instead of relying on diet alone. I am well aware that crash diets do not work […] I need to find something that offers more. (Lene)

In Lene’s narrative, the initiation of plans for action is a deeply reflective exercise, marked by an internal debate and the consideration of multiple variables. This might be the case because her need frustration was due to internal factors. She engages in a thoughtful process that incorporates concrete information. For example, her statement, “I am well aware that crash diets do not work,” shows that her initiation of plans for action is informed by specific objective knowledge about the ineffectiveness of certain approaches to weight loss. Additionally, her initiation of plans for action involves a complex interplay of variables–her weight, her health, her appearance, and her past experiences with dieting and exercise. She even acknowledges the tension between her logical, reasoned plans for exercise and some unidentified but powerful barriers that prevent her from acting on those plans. The culmination of her initiating plans for the action phase is a specific intention, articulated as “I want to focus on exercise instead of relying on diet alone.” This intention represents her commitment to a particular course of action, but it also highlights the challenges she faces in translating this intention into sustained action.

### 3.4 Action

The action phase of the restoration process involved the specific steps participants took to cope with the need frustration and unfulfillment and restore their psychological needs. Broadly, the strategies were classified into two categories. The first strategy involved stopping or avoiding the behavior. The other strategy was characterized by actively working against the challenges, fighting back. Rather than withdrawing, the participants who applied this strategy engaged in actions to directly address and overcome the obstacles to restore their needs. The first strategy was typically applied by participants who experienced need frustration due to the actions of others, while the second strategy was typically applied by participants who experienced need unfulfillment or need frustration due to internal factors.

Delving deeper into the first strategy, instances of need frustration due to the actions of others often prompted participants to either avoid or stop the behavior altogether. For example, Laura, who had been sidelined at her team gym team, stopped her participation, “Then I stopped.” In contrast, Lisbeth’s approach within the action phase represents a slightly different strategy for need restoration after incidences of need frustration in physical education. Her strategy represented an avoidance tactic: “I always sought to avoid participation by claiming to be in pain or that something else was wrong. “Unlike Laura’s direct decision to withdraw, Lisbeth’s action involved creating scenarios that justified her non-participation. The key difference between Laura and Lisbeth’s responses in the action phase of their psychological need restoration process lies in the nature of their participation in physical activity – voluntary for Laura and obligatory for Lisbeth. Laura, in a voluntary participation context, directly ceased participation, effectively removing herself from the source of need frustration. In contrast, Lisbeth, within an obligatory context, applied indirect avoidance strategies, like feigning illness, to navigate the constraints of obligatory participation and restore her psychological needs. This contrast was a general pattern in the data, highlighting how the voluntary or obligatory nature of participation influences individuals’ pathways for need frustration in physical activity.

Further, the participants who experienced need frustration in a voluntary activity context tended to take up other types of physical activity at a later point, whereas participants who had experienced need frustration in an obligatory physical activity context tended to avoid sport and exercise settings for extended periods. Lisbeth, for example, at 69 years old, continued to avoid these activities after her incidences of need frustration in physical education that occurred 54 to 63 years earlier. Similarly, another participant, Niels (aged 68), narrated:

I have never really been the athletic type and was the one who got picked last in physical education classes […] That has caused some emotional issues over the years […] It is something that today… I think it has shaped my attitudes toward sports and physical activity ever since […] I found my sense of achievement elsewhere.

In contrast, Laura decided to participate in fitness classes shortly after she stopped her voluntary team gym practice.

Shifting the focus to the second category of coping strategies, fighting back, this strategy was evident in participants dealing with need unfulfillment and participants whose experience of need frustration is due to internal factors. A common way of fighting back from experiences of need unfulfillment and need frustration due to internal factors was to make different kinds of adjustments. These ranged from minor adjustments, such as making slight tweaks in routines or enhancing existing skills through additional training or resources within their current environment, to more salient adjustments, such as changing their sport or environment. An example of someone who chose the latter strategy was Andreas, who experienced psychological need unfulfillment in a football club environment. He acted by switching clubs. He said, “I decided to switch clubs […] I quickly felt like a part of the team. This gave me a renewed sense of purpose and community in the sport.” Andreas’ proactive decision to switch clubs was a significant adjustment in response to the need unfulfillment he experienced. Upon joining the new team, Andreas experienced a quick integration, feeling like a part of the team. Thus, his needs were successfully restored. Another example of fighting back after incidences of need unfulfillment stems from Mathilde (aged 21), who did not feel a part of the community on her swim team because she chose not to participate in competitions. In response, she switched to a new sport, explaining:

So, I chose that I did not want to swim anymore [...] Then I started strength training with friends. I wanted to look big and strong. Sculpt my body [...] It was something I could control myself. There was no one to tell me what to do.

Similarly, Lene, who experienced need frustration due to internal factors, feeling bad when looking in the mirror because she had gained weight and thus intended to start exercising, said: “Now I have signed up for a gymnastics team.” By choosing to exercise more, she is taking control of her situation, actively working toward improving an aspect of herself that she is dissatisfied with, thus trying to move her need state toward satisfaction. However, changing behavior was not the only strategy she needed to apply. Alongside, Lene also shifted her mindset about physical activity. She acknowledged, “It is something that lies in my consciousness as a duty. But it is primarily associated with pleasure because I feel so good afterward.” This cognitive adjustment helped her focus on the positive outcomes of her activities, maintaining her engagement and commitment. The combination of behavioral change and cognitive reframing thus enabled Lene to align her actions with her values and achieve a more fulfilling engagement with physical activity.

## 4 Discussion

We offer the first qualitative investigation of the psychological need restoration process in the SDT literature. Below, we describe our main findings within physical activity contexts, how they advance the extant literature and have implications for a more dynamic representation of psychological needs in future SDT models. We also offer several directions for future research.

Our primary aim is to explore the psychological need restoration process within different physical activity contexts. Psychological need restoration involves addressing discrepancies between actual and desired need states, which can trigger adaptive behaviors to restore balance. While the restoration process has some context-invariant characteristics, its manifestations can vary significantly depending on the specific context (Waterschoot et al., 2020). This study seeks to provide a comprehensive understanding of how individuals experience and respond to need frustration and restoration in physical education, exercise, and sports settings.

The initial phase of the restoration process ‘discrepancies between actual and desired need states’ align with past findings which reported that autonomy need frustration triggered the restoration process (Sheldon and Gunz, 2009; Van Prooijen, 2009; Radel et al., 2011, 2013, 2014). Extending past research, we identify specific factors within PA contexts that contribute to the perception of discrepancies between actual and desired need states, such as physical separation (e.g., Laura feeling sidelined at her team gym, despite her desire for a sense of belonging to the team), conditional inclusion (e.g., Laura perceives her inclusion in the team as dependent on her competencies), and comparative disadvantage (e.g., Lisbeth and Niels, who experienced challenges due to their larger body sizes compared to their peers). These factors provide granular insights into the various factors that can initiate the restoration process within PA contexts.

The second phase of the restoration process we identified, ‘experiencing negative emotions’, is not adequately addressed in the existing restoration process literature. Our findings suggest that experiencing negative emotions provides an interpretive layer that refines the initial perceptions of discrepancies between actual and desired need states. These results align well with the theoretical framework of cybernetic control processes posited by Carver and Scheier (1990, 2012), underlining the significance of feedback mechanisms in behavioral self-regulation. Carver and Scheier propose that negative emotions functions as an integral component of feedback loops, actively guiding modifications in behavior. Rather than being a passive psychological consequence, negative emotions serves as a functional signal, indicating a gap between current social engagement levels and desired social interactions. In our study, we further found that the experience of negative emotions provides an interpretive layer that goes beyond the initial emotion response, directing attention to discrepancies between actual and desired need states. This also resonates with Carver and Scheier’s assertion that negative emotion feedback instigates adjustments in strategies to reduce discrepancies between present circumstances and targeted goals. For instance, feelings of sadness in response to social exclusion not only signal an emotion but also heighten awareness toward potential opportunities for social inclusion, thus guiding individuals toward more focused restorative activities. These arguments also align well with the research of Kramer and Yoon (2007) on approach-avoidance motivation and the use of emotions as information. Their series of studies suggests that emotions, including emotions and mood, provide individuals with critical information about their internal and external environments. For example, someone experiencing negative emotion in a situation might, based on their approach or avoidance motivation, interpret this as a cue to either engage in change-oriented behavior or to avoid potential harm. In conclusion, we advocate for the merits of considering emotion responses as active components in psychological need restoration and behavior change.

The ‘initiating plans for action’ phase was significantly influenced by social dynamics, opportunities, and the resources available to the participants. These insights into the initiation of action plans are in alignment with Carver and Scheier’s feedback theory of self-regulation (1990). Carver and Scheier emphasize the role of external factors, including social interactions, in shaping coping strategies. Such influences are reflected in our observations, where decisions made by participants, such as Andreas’ decision to change clubs, were notably influenced by social connections. Additionally, the adaptability demonstrated by participants, like Hanne’s utilization of available resources for tennis coaching, resonates with Carver and Scheier’s perspective on how environmental factors influence behavior. In essence, our study’s insights into the role of social and environmental factors in the development of action plans underscore the significance of external stimuli in shaping effective restoration strategies. We further found that the phase of initiating plans for action can manifest in two distinct ways: As a deeply reflective exercise (such as deliberate consideration of future outcomes and weighing of various options) or as a more straightforward, reactionary internal dialog (like an immediate, instinctive response to a current situation). This observation is in agreement with the dual systems outlined in the Reflective-Impulsive Model (RIM) by Strack and Deutsch (2004). While much of the existing literature, such as the studies by Radel et al. (2011, 2013, 2014), focuses on the role of automatic, impulsive systems in initiating restoration activities, our findings suggest that the process of initiating plans involves not only these automatic impulses but also a conscious reflective layer. The culmination of this phase is a specific intention, setting the stage for action. The ‘initiating plans for action’ phase complements and extends the existing literature on the restoration process, particularly focusing on the level of awareness and cognitive appraisal that individuals undergo.

The ‘action’ phase in the restoration process, as identified in our study, intricately involves how participants cope with different types of need frustrations (those due to external contingencies or internal factors) or need unfulfillment. By referring to the Approach and Avoidance Behavior framework (Corr, 2013), which categorizes responses into the Behavioral Approach System (BAS), the Fight-Flight-Freeze System (FFFS), and the Behavioral Inhibition System (BIS), we can gain a deeper insight into these varied coping mechanisms. In situations where need frustration arises from external factors, such as restrictive rules or unsupportive environments, our study observed a predominant ‘flight’ response. This defensive strategy, characterized by avoidance or withdrawal, aligns with the activation of the FFFS. It signifies an immediate reaction to minimize discomfort or harm by distancing oneself from the source of frustration. This reaction is consistent with findings by Maner et al. (2007), DeWall et al. (2009), Radel et al. (2014), and Fang et al. (2017, 2018, 2021, 2022), who noted that such withdrawal is a common strategy to circumvent further frustration. However, when the perceived threats from these external contingencies are less immediate or severe, the BIS might dominate, leading to more cautious and considered approach behaviors. This shift from an immediate flight to a strategic reassessment of the situation is a critical aspect of the need restoration process that the Approach and Avoidance Behavior framework (Corr, 2013) helps elucidate. In voluntary PA contexts like sports clubs, this may manifest as participants choosing to leave frustrating activities, thus exercising their autonomy to seek fulfillment in alternative activities.

The action tendencies in response to need unfulfillment (e.g., lack of available opportunities and challenges to become better) or frustration due to internal factors (e.g., increased weight reduces capability for specific tasks) present a different scenario. Rather than withdrawing, individuals are more likely to engage in a ‘fight’ response, characterized by actively seeking new opportunities (e.g., shifting to a team or activity that better supports their needs) or making adjustments (e.g., cognitive adjustments or investing in lessons to improve skills). This proactive approach is indicative of a BAS activation. This finding resonates with Sheldon and Gunz’s (2009) assertion that deficiencies in psychological needs drive individuals to seek experiences that will fulfill such needs. Thus, our study delineates the distinct pathways of coping with need frustration due to external contingencies, need frustration due to internal factors, and need unfulfillment, which previous literature has not differentiated.

The results demonstrate the different approaches to restoring needs after incidences of need frustration in voluntary and mandatory contexts, highlighting the importance of context. The need restoration process can be viewed as a form of coping strategy following need frustration. Waterschoot et al. (2020) emphasize that when individuals face need frustration, they engage in deliberate behaviors aimed at alleviating this frustration and restoring need satisfaction. This aligns with broader coping mechanisms that involve adapting to environmental challenges and managing stressors. The process of restoring psychological needs, for example, involves seeking new opportunities, engaging in skill development, and modifying one’s environment to better support need satisfaction. Similarly, our findings suggest that participants employed various coping strategies–whether it be changing clubs, seeking social connections, or utilizing available resources–to manage their psychological needs in both voluntary and mandatory physical activity contexts. Therefore, the need restoration process can be considered a proactive and dynamic coping mechanism that individuals use to navigate and mitigate need frustration.

## 5 Strengths and limitations

This study adds to the growing literature on the process of psychological need restoration in multiple ways. First, we identified four stages in the restoration process–discrepancies between actual and desired need states, experiencing negative emotions, initiating plans for action, and action. Previous studies have not examined the dynamic nature of need restoration in such a nuanced way. Second, we looked at the restoration of all three psychological needs proposed by SDT using rich qualitative data; previous studies have been quantitative in nature, and some of them focused on one psychological need only. Third, we distinguished between incidents of need frustration and need unfulfillment and found differences in how the participants restored their needs depending on whether they had prior experience of need frustration versus need unfulfillment. Fourth, previous studies have primarily investigated early-stage adaptions after incidences of need frustration (DeWall et al., 2009; Radel et al., 2011; Fang et al., 2018, 2022). The present study has also explored repeated or long-term need frustration, which, as indicated by Fang et al. (2018, 2022), has not been studied in past research. Taken together, our findings can be used in the future by SDT researchers to develop theoretical models that are not static but acknowledge both the dynamic nature of psychological needs as well as their multidimensionality (satisfaction, frustration, and unfulfillment).

Our study also offers key insights for PA understanding and promotion. Firstly, it identifies specific factors, like physical separation and conditional inclusion, that trigger the restoration process. This knowledge can improve strategies to address need frustration in PA contexts. The study highlights the role of negative emotions in signaling discrepancies and guiding behavioral changes, in line with Carver and Scheier’s cybernetic control process theory. Recognizing the functional role of negative emotions can enhance PA interventions by using emotional responses as cues for behavior change. Additionally, the study shows that the initiation of action plans is significantly influenced by social dynamics and available resources, underscoring the importance of supportive environments in facilitating physical activity. Finally, the study’s insights into the ‘action’ phase, using the Approach and Avoidance Behavior framework, reveal distinct responses to different types of need frustrations. This differentiation is crucial for tailoring PA interventions to address specific challenges, enhancing engagement and sustained participation in PA.

However, despite its strengths, the study is not without its limitations. The focus on Danish adults might limit the broader applicability of our findings. Cultural, social, and economic nuances specific to Denmark could potentially influence the experiences of our participants, and these might not seamlessly translate to other demographic or cultural contexts (Flick, 2015). The focus on physical activity as behavior might limit the applicability of our findings to other types of behavior. Physical activity, with its unique determinants (see Bauman et al., 2012), may not represent or mirror the complexities and nuances of other behaviors. Additionally, the retrospective nature of the narratives in our study might introduce elements of recall bias. The accuracy of participants’ memories or their present emotional states could inadvertently color their past experiences, thereby influencing the narratives they shared (Schwarz and Sudman, 2012). Further, we employed interview techniques to prompt deeper participant insights into their experiences of need frustration and restorative adaptations. While acknowledging that interviews influence participants’ conceptualization of their experiences (Loftus and Palmer, 1974; Brinkmann and Kvale, 2018), we suggest that the increased richness of data generated might compensate for this limitation.

### 5.1 Future directions

In terms of future research, it could potentially include diverse behaviors (e.g., other health behaviors, learning behaviors), settings (e.g., families, classrooms, health clinics, and workplaces), methodologies (e.g., ecological momentary assessments), and populations to render a more holistic understanding of the restoration process. For example, in terms of diversity in settings, the predominance of laboratory-based studies in this field calls for an expansion into real-life settings. Future research could also extend the focus to diverse population groups, such as adolescents, the elderly, patient groups, or employees. This approach would offer more diverse perspectives of where the restoration process might play out differently, influenced by diverse real-world complexities. In terms of methodologies, intensive longitudinal designs could allow researchers to examine the unfolding of the restoration process repeatedly over days or weeks and investigate successful and unsuccessful restoration attempts. Such research designs would provide just-in-time insights into the emotions and cognitive states that act as triggers for the restoration process, thereby minimizing the limitations associated with retrospective accounts. The effects of social agents on the restoration process could also be examined, for instance, the role of need supportive social agents.

Further, future research should examine the restoration processes separately for need frustration due to external contingencies, need frustration due to internal factors, and need unfulfillment. The current literature is heavily focused on the restoration process after incidences of need frustration due to external factors (e.g., Radel et al., 2014; Fang et al., 2018, 2021), overlooking other restoration pathways. Mapping such diverse pathways, potentially by also incorporating relevant literature on goal self-regulation and cybernetics (Carver and Scheier, 1990, 2012), can offer a more comprehensive understanding of the different facets of psychological need restoration.

## Data availability statement

The raw data supporting the conclusions of this article will be made available by the authors, without undue reservation.

## Ethics statement

The studies involving humans were approved by SDU Research and Innovation Organization. The studies were conducted in accordance with the local legislation and institutional requirements. The participants provided their written informed consent to participate in this study.

## Author contributions

BD: Conceptualization, Data curation, Formal analysis, Investigation, Methodology, Project administration, Writing – original draft, Writing – review & editing. NN: Conceptualization, Formal analysis, Resources, Supervision, Validation, Writing – original draft, Writing – review & editing. KE-Ø: Conceptualization, Funding acquisition, Methodology, Project administration, Supervision, Validation, Writing – review & editing. TB: Conceptualization, Methodology, Project administration, Resources, Supervision, Validation, Writing – review & editing.
