# Supplementary material for: Exploring the process of restoring psychological needs after incidences of frustration and need unfulfillment
Source: Front Psychol. 2024 Jun 4;15:1413963. doi: 10.3389/fpsyg.2024.1413963 (PMC11188414; doi:10.3389/fpsyg.2024.1413963)
Supplement: Supplementary file 1 [file Data_Sheet_1.docx]

Supplementary Material

# Participant characteristics

This supplementary table presents detailed participant characteristics, including the participant pseudonyms used in the manuscript, gender, age, physical activity frequency level, interview form, interview location, and interview length, ensuring transparency and aiding in understanding the study's participant diversity and context.

| Pseudonym | Gender | Age | Physical activity frequency  level | Interview form | Interview location | Interview length (minutes) |
| --- | --- | --- | --- | --- | --- | --- |
| Alberte | Female | 27 | Average | Face-to-face | Participants’ home | 118 |
| Anders | Male | 28 | Low | Face-to-face | Participants’ work place | 104 |
| Andreas | Male | 16 | Average | Face-to-face | University meeting room | 112 |
| Anette | Female | 72 | High | Face-to-face | Participants’ home | 162 |
| Bjarne | Male | 60 | High | Face-to-face | Participants’ home | 167 |
| Camilla | Female | 17 | High | Face-to-face | Participants’ home | 145 |
| Charlotte | Male | 52 | Average | Face-to-face | Participants’ home | 162 |
| Dagmar | Female | 75 | Average | Face-to-face | Participants’ home | 132 |
| Elvira | Female | 75 | Average | Face-to-face | Participants’ home | 140 |
| Emil | Male | 21 | Average | Face-to-face | Participants’ home | 89 |
| Erik | Male | 65 | Average | Face-to-face | Participants’ home | 138 |
| Flemming | Male | 51 | Low | Face-to-face | Participants’ home | 99 |
| Frederik | Male | 28 | High | Online | - | 138 |
| Hanne | Female | 75 | Low | Face-to-face | Participants’ home | 90 |
| Ingemarie | Female | 71 | High | Face-to-face | Participants’ home | 142 |
| Jens | Male | 58 | Low | Face-to-face | Participants’ home | 124 |
| Julie | Female | 28 | Low | Online | - | 81 |
| Karen | Female | 64 | Average | Online | - | 144 |
| Kasper | Male | 34 | Average | Face-to-face | Participants’ work place | 168 |
| Kristine | Female | 31 | Low | Face-to-face | Participants’ home | 88 |
| Lars | Male | 51 | High | Face-to-face | Participants’ home | 109 |
| Laura | Female | 17 | Low | Face-to-face | Participants’ home | 77 |
| Lene | Female | 66 | Low | Face-to-face | Participants’ home | 162 |
| Linda | Female | 60 | High | Face-to-face | Participants’ home | 112 |
| Lis | Female | 79 | Average | Face-to-face | Participants’ home | 162 |
| Lisbeth | Female | 69 | Low | Face-to-face | Participants’ home | 94 |
| Louise | Female | 26 | Average | Face-to-face | Participants’ home | 155 |
| Maria | Female | 41 | Low | Face-to-face | Public park | 95 |
| Marie-Louise | Female | 40 | Low | Face-to-face | Participants’ home | 170 |
| Tobias | Male | 24 | Average | Face-to-face | Participants’ home | 108 |
| Mathilde | Female | 21 | Low | Face-to-face | Participants’ home | 114 |
| Mette | Female | 30 | High | Face-to-face | Participants’ home | 106 |
| Mogens | Male | 70 | High | Face-to-face | Participants’ home | 151 |
| Mona | Female | 65 | Low | Face-to-face | Participants’ home | 142 |
| Niels | Male | 68 | Average | Face-to-face | Participants’ home | 110 |
| Ole | Male | 72 | High | Face-to-face | Participants’ home | 142 |
| Per | Male | 52 | Low | Face-to-face | Participants’ home | 149 |
| Poul | Male | 79 | Average | Face-to-face | Participants’ home | 178 |
| Svend | Male | 69 | Low | Face-to-face | Participants’ home | 122 |
| Søren | Male | 29 | Low | Face-to-face | Library | 132 |
| Tobias | Male | 20 | High | Online | - | 82 |
| Trine | Female | 61 | High | Face-to-face | Participants’ home | 137 |

1. **Interview guide**

The interview guide for this study is presented below to ensure transparency in the research process. It includes the researcher's introduction, the content of the interview, guarantees of anonymity, statements regarding participant volition, demographic inquiries, exploration of significant life chapters, questions about behaviour and motivation, and procedures followed after the interview.

| Theme | Content / Question |
| --- | --- |
| Presentation of researcher | XXX |
| Presentation of project | XXX The project aims to gain new insights into adults' movement habits, as well as the influences of motivation, motives/barriers and opportunities on various forms of movement.  You have already contributed significantly to the study with your participation in the survey. Then you have been so kind to agree to participate in this follow-up interview as well. Thank you. |
| Presentation of the content of the interview | Today, I will ask you to tell me your life story, and we will talk about your physical activity habits and motivation within life transitions and lifestyle contexts.  There are no right or wrong answers. I am interested in your life and your experiences. |
| Time frame | The interview will last approximately [?] hours.  I would like to audio record the interview on two Dictaphones. The recordings are only used internally for the purposes of the analysis. Is that okay with you? |
| Audio recording |  |
| Anonymization | Your statements will be de-identified so that people who are not very familiar with you and your life story can't associate them with you. |
| Volition | It is voluntary for you to participate, and you can withdraw your consent. |
| Questions | Do you have any questions or comments about the research project or interview? |
| Consent | Before we begin the interview, I would like to ask: Do you consent to participate in the interview? AND: May I have your consent to use the information and statements you give me in this interview in the papers I will write? |
| Demography | Will you start out by telling me a bit about yourself?  Name, Age, Education, Employment, Family |
| Chapters of life | Now, we will start focusing on your life story.  All people's lives can be written down in a book. I would like you to think about your life as if we were writing a book about it. First, I want to ask you to think about the different chapters in your book. I have a piece of paper here that can help you out. The first column contains the years of your life - from zero, the day you were born, until today.  If you start from the day you were born, when does the first chapter end? Put a line there and name the chapter.  Then you move on to the next chapters of your life and mark where they start and end, and name the chapter until you reach your current age.  You are free to use the number of chapters that suits your life.  Now, we will take a closer look at each of your chapters. The focus will be on:   1. How you moved 2. How you were motivated   The impact of the social context in which you lived. |
| Behavior questions | How did you do in this chapter of your life?  Did you do any physical activity during this chapter of your life?  Why did you [activity]?  Was it normal to [activity] where you lived?  How did it influence your motivation to [activity]?  Who introduced [activity] for you?  Tell me about how it happened?  Whose choice was it, that you [activity]?  How did it influence your motivation to [activity]?  Do you think that, doing [the activity] reflected what YOU wanted and who YOU where?  How?  What did your family and friends think about your engagement in?  How did it influence your motivation to [activity]?  Did you feel skilled at [activity]?  How did it influence your motivation to [activity]?  Do you recall any specific episodes or memories from [activity] during this period? Tell me about it.  Why did you stop? |
| Comments to add | Do you have any comments to add? |
| Thanks | Thank you for your story, and answers on my questions. |
| And now… | What will happen now is that the audio recording will be transcribed and then the interview will subsequently be analyzed. The analysis will result in articles which hopefully will be published in international scientific journals.  XXX  Otherwise, feel free to contact us later if you have questions, comments, or concerns. |

1. **Code matrix**

This supplementary table includes a code matrix from the framework analysis, which links the participants’ pseudonyms with states of psychological needs (frustration, unfulfillment, and satisfaction) and their respective restoration adaptations. This matrix provides clarity on the analytical and interpretative processes used in the study.

| Pseudo-nyms | Perceived need frustration | | | Perceived need unfulfillment | | | Perceived need satisfaction | | | | Restoration adaptations | | |
| --- | --- | --- | --- | --- | --- | --- | --- | --- | --- | --- | --- | --- | --- |
|  | Need for autonomy | Need for competence | Need for relatedness | Need for autonomy | Need for competence | Need for relatedness | Need for autonomy | Need for competence | Need for relatedness | |  | | |
| Alberte |  |  |  |  |  |  |  | | |  | |  |  |
| Anders |  |  |  |  |  |  |  | | |  | |  |  |
| Andreas |  |  |  |  |  |  |  | | |  | |  |  |
| Anette |  |  |  |  |  |  |  | | |  | |  |  |
| Bjarne |  |  |  |  |  |  |  | | |  | |  |  |
| … |  |  |  |  |  |  |  | | |  | |  |  |
